# Graft revascularization is essential for non-invasive monitoring of transplanted islets with radiolabeled exendin

**DOI:** 10.1038/srep15521

**Published:** 2015-10-22

**Authors:** Wael A. Eter, Desirée Bos, Cathelijne Frielink, Otto C. Boerman, Maarten Brom, Martin Gotthardt

**Affiliations:** 1 Department of Radiology and Nuclear Medicine, Radboud University Medical Center, Nijmegen, The Netherlands

## Abstract

Islet transplantation is a novel promising strategy to cure type 1 diabetes. However, the long-term outcome is still poor, because both function and survival of the transplant decline over-time. Non-invasive imaging methods have the potential to enable monitoring of islet survival after transplantation and the effects of immunosuppressive drugs on transplantation outcome. ^111^In-labeled exendin-3 is a promising tracer to visualize native and transplanted islets by SPECT (Single Photon Emission Computed Tomography). In the present study, we hypothesized that islet microvasculature plays an important role determining the uptake of exendin-3 in islets when monitoring transplant survival. We observed ^111^In-exendin-3 accumulation in the transplant as early as three days after transplantation and an increase in the uptake up to three weeks post-transplantation. Islet-revascularization correlated with the increase in ^111^In-exendin-3 uptake, whereas fully re-established islet vasculature coincided with a stabilized uptake of the radiotracer in the transplant. Here, we demonstrate the importance of islet vasculature for *in vivo* delivery of radiotracers to transplanted islets and we demonstrate that optimal and stable uptake of exendin four weeks after transplantation opens the possibility for long-term monitoring of islet survival by SPECT imaging.

Reversal of type 1 diabetes by transplanting pancreatic islets has emerged as a promising therapeutic approach^1^,^2^. However, insulin independence shows a rapid decrease and can hardly be preserved beyond 5 years after the intervention^3^. Moreover, several studies reported impaired islet survival after liver transplantation, which was caused by insufficient revascularization^4^ and triggering of blood-mediated inflammatory reactions^5^, along with lipo and/or gluco-toxicity^6^,^7^.

Current functional tests cannot determine whether graft failure is caused by functional impairment of β-cells or β-cell loss, as many factors can be involved in this process (i.e. immune rejection, decline in islet function through metabolic or inflammatory stress)^3^,^8^. Biomedical imaging techniques can be used to monitor islet survival in addition to islet function measured by clinical testing and can therefore contribute to future clinical decision making regarding treatment assignment or adaptation. Pre-labeling of islets with super-paramagnetic iron oxide particles (SPIOs) for MR imaging of islet transplants has been introduced a decade ago^9^. Further studies demonstrated that human islets transplanted in rodents can be monitored and quantified by MRI up to 6 months post-transplantation^10^. Labeling of the islets with SPIOs prior to transplantation is therefore a promising technique for clinical studies^11^.

In other studies, radioactive-based imaging methods were used for their advanced detection sensitivity of *in vivo* tracer uptake by transplanted or native islets^12^,^13^. In particular, SPECT imaging after injection of ^111^In-labeled exendin was reported as a promising strategy to non-invasively visualize engrafted islets, as well as to visualize and quantify BCM in the pancreas of healthy and diabetic subjects^14^,^15^. Therefore, this tracer could be suitable for repeated longitudinal clinical imaging of islet transplants. The advantage of the tracer is the ability of repeated scanning without requiring pre-labeling of the islets prior to transplantation. However, the targeting properties of the radiotracer to the islet grafts early after transplantation may depend on the vasculature that is disrupted after islets isolation^16^,^17^,^18^.

Imaging of islet grafts in the skeletal muscle has been demonstrated previously^15^, and allows monitoring of the transplant since the islets remain localized in a small engraftment area, where specific uptake can be easily discriminated from the surrounding tissue. Making use of this transplantation model, we determined the correlation between islet graft revascularization and the uptake of ^111^In-exendin-3 in the transplant for several weeks following islet transplantation.

## Results

### Radiolabeled exendin-3 binds to the GLP-1R on mouse islets *in vitro*

**Figure 1:**
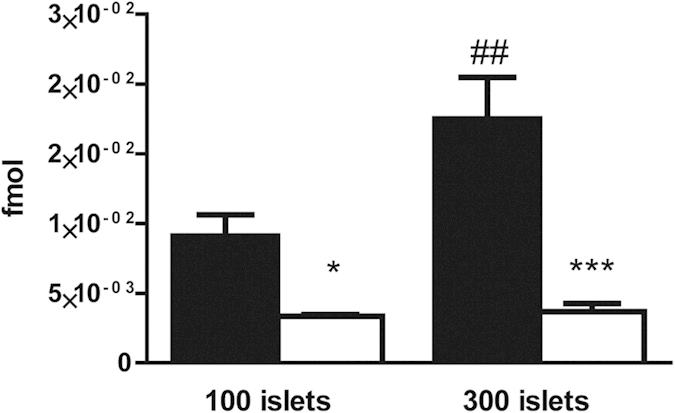
*In vitro* binding of radiolabeled exendin-3 to mouse pancreatic islets. Quantification of radiolabeled exendin-3 (black columns) shows higher binding to 300 islets than to 100 islets. Blocking of the receptor with an excess of unlabeled exendin-3 (white columns) significantly decreased the binding. Data are shown as means ± SEM from 3 experiments. *p < 0.05, ***p < 0.001 compared with the corresponding amount of islets, whereas *##p* < 0.01 compared with 100 islets.

Incubation of 1.5 fmol of radiolabeled exendin-3 with 100 islets during 4 hours resulted in the binding of 9.1 × 10^−3^ fmol (Fig. 1). Binding of radiolabeled exendin-3 was reduced to 3.3 × 10^−3^ fmol in the presence of excess of unlabeled exendin-3 (*p* < 0.05). Binding of radiolabeled exendin-3 was two-fold higher when added to 300 islets (17.5 × 10^−3^ fmol) and binding was successfully decreased to 3.7 × 10^−3^ fmol by applying an excess of unlabeled exendin-3 (*p* < 0.001), which confirmed the expression of the GLP-1R.

### Exendin-3 uptake in the skeletal muscle is localized in the transplanted islets

**Figure 2:**
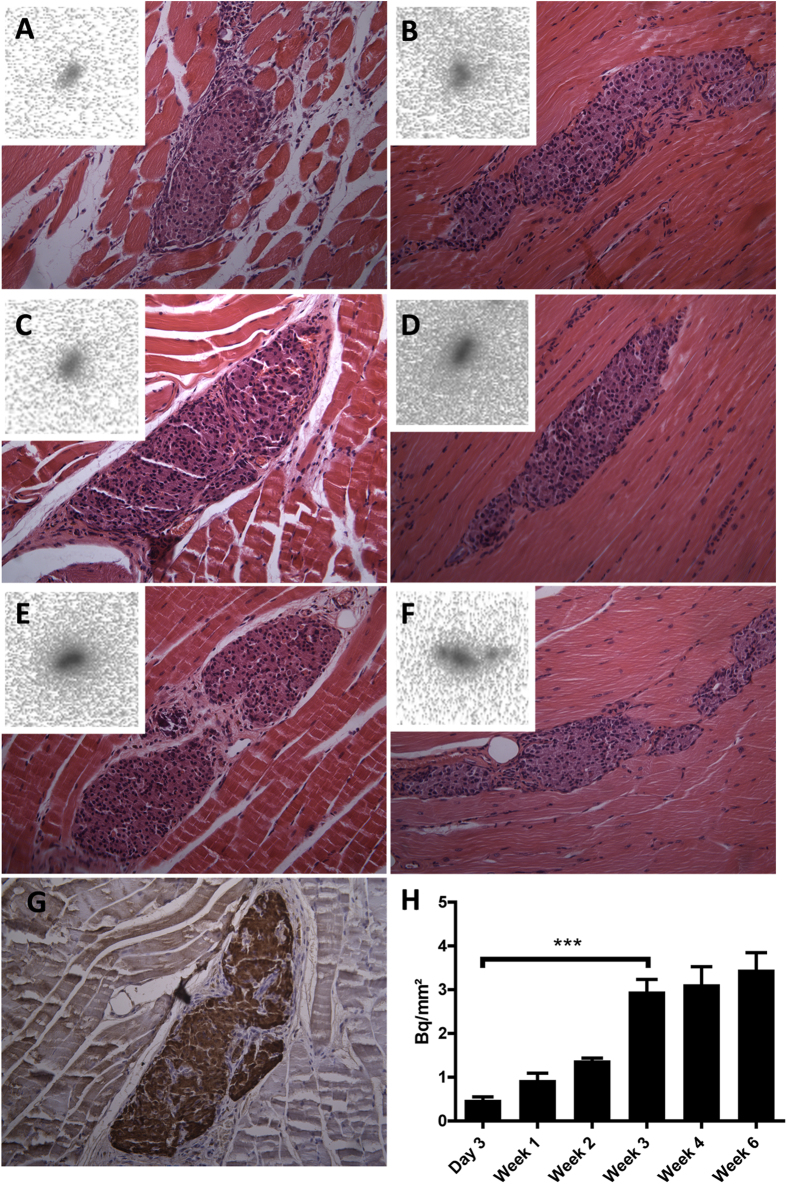
*Ex vivo* measurement of exendin-3 uptake by transplanted islets. (A–F) Autoradiography of muscle sections shows hotspots of In-111 accumulation. Histochemical staining of the corresponding autoradiography section shows co-localization between the uptake of exendin-3 and the transplant. (**G**) Adjacent sections were stained with anti-insulin mAb to confirm the presence of β-cells (brown). (**H)** Quantification of In-111 uptake (expressed in becquerel/mm^2^) according to autoradiography shows significant increase between 3 days to 3 week-old transplants (***p < 0.001) and stabilized uptake between 3 and 6 weeks post-transplantation.

**Figure 3:**
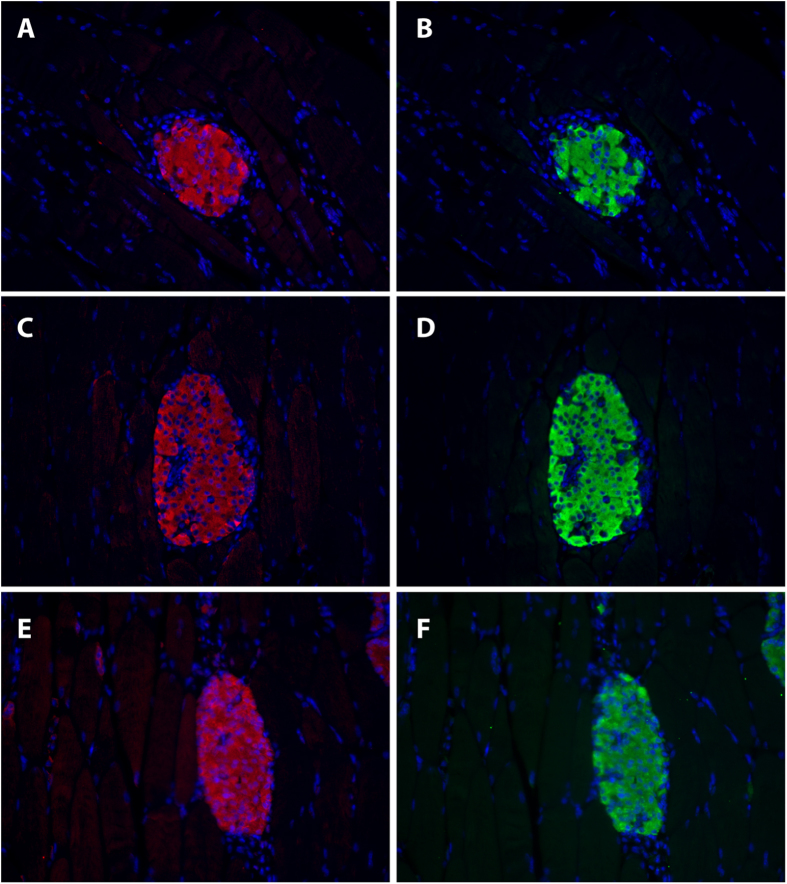
GLP-1R expression is maintained in the islets early after transplantation. Representative images of 3 days (**A–B**), 2 weeks (**C–D**) and 4 week-old transplants (**E–F**) stained for GLP-1R (red) and insulin (green).

In order to determine whether the uptake of exendin-3 originates from the transplant within the skeletal muscle, we performed *ex vivo* autoradiography of the muscle regions that contained the islets, followed by histochemical staining. As evidenced in Fig. 2(A–F), there was accumulation of ^111^In-exendin-3 within well-localized regions of the muscle after 3 days, 1, 2, 3, 4 and 6 weeks in the islet transplants. Sections were next analyzed by HE staining, where the uptake was confirmed as islet-specific. Immunohistochemical staining of muscle sections with an anti-insulin monoclonal antibody confirmed that the transplant predominantly consisted of β-cells (Fig. 2G). Quantitative analysis of ^111^In-exendin-3 accumulation within transplanted islets by autoradiography was 0.48 ± 0.068, 0.99 ± 0.16, 1.35 ± 1.08, 2.97 ± 0.27, 2.73 ± 0.4 and 3.45 ± 0.34 Bq/mm^2^ at 3 days and 1, 2, 3, 4, 6 weeks after transplantation, respectively (Fig. 2H). Immunostaining of the GLP-1R with a polyclonal antibody confirmed the expression of the receptor in the transplants (Fig. 3).

### The density of newly formed blood vessels in the transplant correlates with the uptake of exendin-3

**Figure 4:**
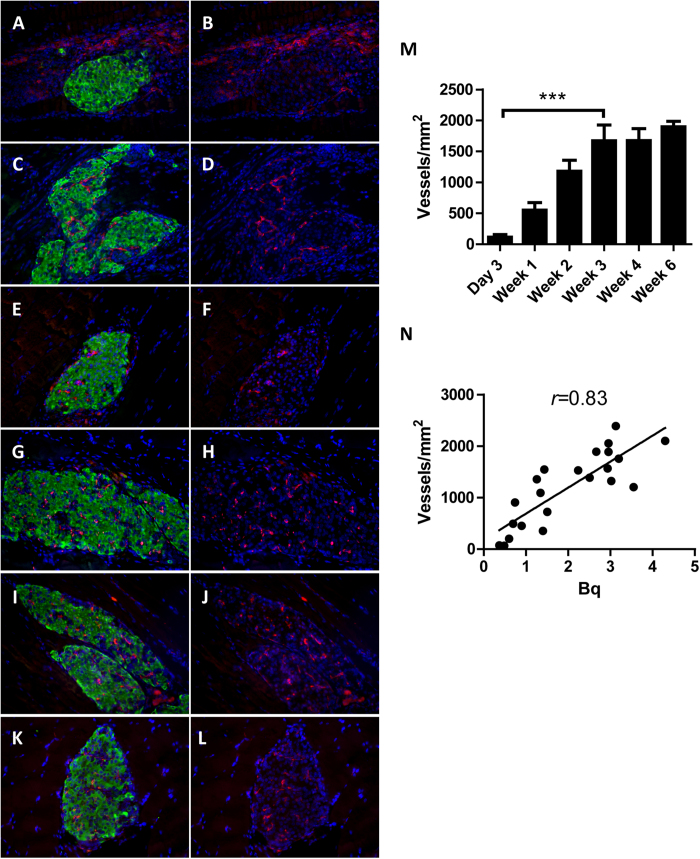
Histological analysis of islets revascularization in the skeletal muscle. (**A–L**) Representative images of 3 days to 6 week-old islet transplants for which β-cells were stained with anti-insulin (green) and the ingrowing VEGFR2-expressing capillaries (red). All sections were counter-stained with DAPI (blue). (**M**) Quantification of vascular density within the insulin positive area shows significant increase in number of vessels from 3 days to 3 weeks (***p < 0.001). ***N**:* Initiation and increase of islets vascular density correlates with increasing exendin-3 uptake (pearson *r* = 0.83). Data are shown as means ± SEM from 4–5 animals.

To investigate whether revascularization of transplanted islets affects the uptake of radiolabeled exendin-3, VEGFR-2 and insulin expression was quantified to determine islet microvasculature and β-cells area, respectively (Fig. 4). The first vascular structures in the graft were observed 3 days after transplantation. At that time, VEGFR-2 positive endothelial cells were mostly surrounding the transplant (Fig. 4A,B). There was notable progress of angiogenesis one week after transplantation, as more endothelial cells had infiltrated the islets (Fig. 4C,D). Intra-islet vasculature was more prominent between 2 weeks and 6 weeks after transplantation, as vessels clearly had penetrated from the periphery into the core of the transplant (Fig. 4E–L). Quantitative analysis of revascularization showed notable progress between 3 days and 3 weeks (*p* < 0.001) and a stablized vascular density between 3 and 6 weeks (Fig. 4M).

First signs of revascularization coincided with low exendin-3 uptake by 3-day old grafts and increase in islets vascular density correlated with the uptake of exendin-3 by the islets (Pearson correlation coefficient *r* = 0.83) (Fig. 4N).

### *In vivo* visualization of islet transplants is dependent on islet revascularization

**Figure 5:**
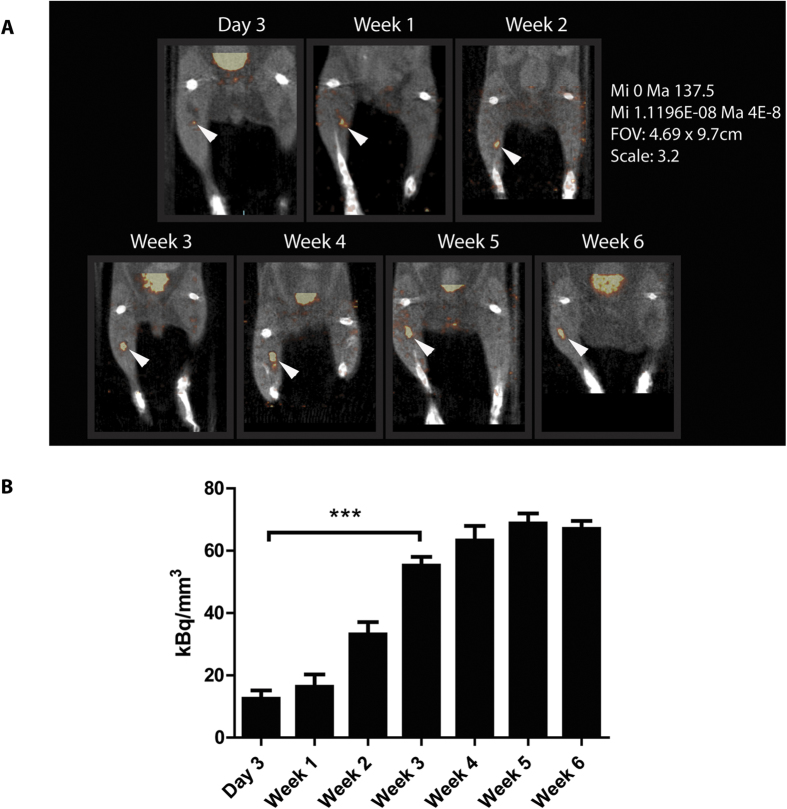
*In vivo* detection of pancreatic islets transplanted in the calf muscle. (**A**) Representative images of C3H mice with transplanted islets. Signal was detected 3 days, 1, 2, 3, 4, 5 and 6 weeks after transplantation (white arrows). (**B**) Quantitative analysis of the SPECT images showed a significant increase in the uptake of ^111^In-exendin-3 between 3 days and 3 weeks post-transplantation (***p < 0.001) and a stabilized uptake between 3 and 6 weeks. Data are presented as kBq/mm^3^ and are normalized by the injected dose.

**Figure 6:**
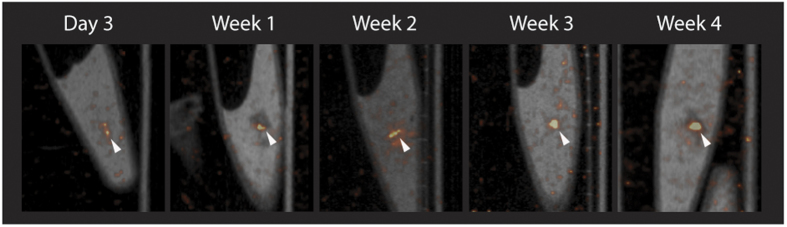
*Ex vivo* detection of pancreatic islets transplanted in the calf muscle. Representative images from *ex vivo* scans of dissected muscles containing the transplant.

**Figure 7:**
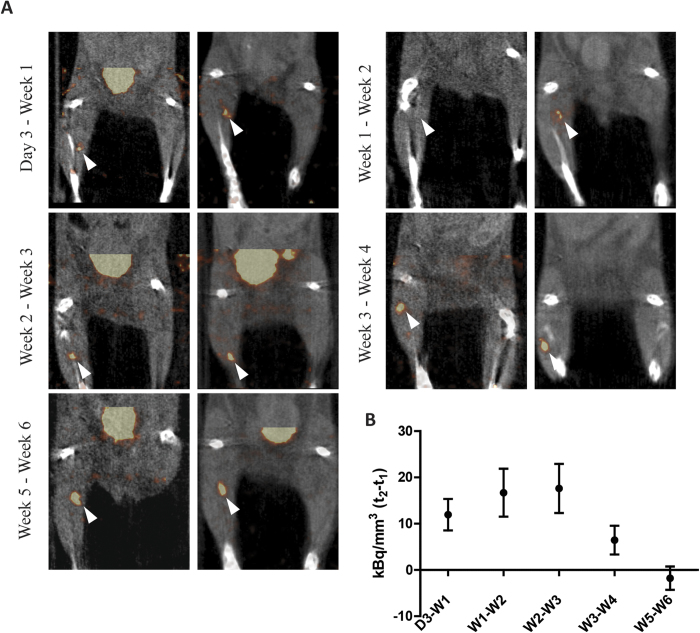
*In vivo* detection of pancreatic islets at different weeks. (**A**) Representative images from *in vivo* scans at two different time-points with one week interval. Transplants are indicated with white arrows. (**B**) SPECT quantitative analysis where t_2_-t_1_ corresponds to 7 days interval between both scans shows improved uptake of ^111^In-exendin-3 and first signs of stabilization of tracer uptake between 3 weeks and 4 weeks post-transplantation.

To assess the correlation between intra-islet vascular density and the tracer concentration in the transplant, SPECT scans were performed at different time-points after transplantation. SPECT visualized 3 day to 6 week-old transplants within the skeletal muscle (Fig. 5A). After 3 days, 12.5 ± 2.7 kBq/mm^3^ accumulated in the transplants, which further increased up to 63 ± 4.7 kBq/mm^3^ at 4 weeks and remained similar up to 6 weeks after transplantation (68 ± 3.2 and 67 ± 2.5 kBq/mm^3^, for week 5 and 6, respectively) (Fig. 5B). *Ex vivo* acquisitions were in line with the *in vivo* data, as signal could be detected after 3 days and was increased over time (Fig. 6). To determine the time-point after which multiple imaging sessions result in reproducible assessment of ^111^In-exendin-3 uptake, each mouse was scanned twice with 7 days of interval, starting 3 days, 1, 2, 3, 4 or 5 weeks after transplantation (Fig. 7A). Quantitative analysis revealed an increase in the signal 7 days after the initial scans with stabilization being observed between 3 and 4 week-old transplants (Fig. 7B). Increase in uptake was independent from the transplant size, since the uptake was normalized by the volume of the transplant. Two transplants which were not visible 3 days or 1 week after transplantation, both became detectable at the time of the second scan.

## Discussion

In the present study, we examined the influence of islet graft revascularization on the targeting characteristics of radiolabeled exendin-3, a radiotracer with great potential as imaging biomarker for non-invasive assessment of β-cell survival after transplantation. We demonstrated that an increase in vascular density, between 3 days and 3 weeks after transplantation, correlated with an increase in the accumulation of ^111^In-exendin-3 in the transplant. Furthermore, in mice in which no signal could be detected three days or one week after transplantation, islet grafts exhibited a detectable uptake at later time-points where vascularity improved. When the vascular bed had been established after 4 weeks, the uptake of exendin-3 indeed stabilized in repeated scans, indicating that islet revascularization has a direct influence on the targeting of transplanted islets after *in vivo* injection of radiotracers. Imaging of islet transplants with radiotracers will therefore reveal reproducible results starting 4 weeks after transplantation, the time-point after which the vascular bed of the transplants appears to have been established.

These findings indicate that recovery of islet vascular network is a prerequisite for reproducible longitudinal evaluation of viable islet grafts. First, complete restoration of islets vasculature improves the transplant signal-to-background ratio which facilitates the quantitative assessment of exendin-3 uptake. Secondly, reproducible uptake of exendin-3 is a requirement to accurately quantify BCM, and changes in the uptake after vasculature has recovered would most likely reflect β-cell loss.

Angiogenesis in recipients with long history of diabetes might be suboptimal^19^. Furthermore, graft angiogenesis rate is dependent on the transplantation site^20^. Therefore, the optimal time-point after which reproducible imaging results are obtained, might be affected by these parameters. The technique will allow, however, to quantify islet survival, months, if not years after transplantation, where failure in maintaining normoglycemia could occur even after successful engraftment of the islets^3^.

It was previously reported that islets transplanted in liver can be visualized with small-animal PET, where the uptake of ^64^Cu-labeled exendin in the liver of transplanted animals was higher as compared to those of the control animals 12 days after transplantation^12^. Here we report the skeletal muscle as a suitable platform for characterization of tracer targeting properties to the islets. Using this model, we could investigate the role of the revascularization of the transplant on radiotracer uptake, since in this model the revascularization could be easily quantified *ex vivo* by histology. This transplantation site could be further used to characterize the targeting properties of novel β-cell radiotracers and to further evaluate existing ones with the advantage of fast histological evaluation of complete transplants.

In conclusion, this study revealed that islet revascularization is a key physiological process when monitoring islet transplants after *in vivo* targeting of the graft with β-cell tracers. Stable and reproducible uptake of exendin was achieved starting 4 weeks after transplantation, indicating that longitudinal assessment of islet survival by SPECT imaging can reproducibly be performed after this time point.

## Research Design and Methods

### Animals

Female C3H/HeNCrl mice (22–30 g) were purchased from Charles River (Calco, Italy). All experiments were conducted in accordance with Radboud University guidelines on humane care and use of laboratory animals, and were approved by the Animal Ethical Committee of the Radboud University, Nijmegen, The Netherlands.

### Pancreatic islet isolation and transplantation

Pancreatic islets were isolated from 8 week-old female C3H/HeNCrl mice by collagenase digestion method. Mice were euthanized and 2 ml of cold RPMI 1640 (Invitrogen, Carmarillo, CA, USA) containing collagenase-V (0.9 mg/ml, Sigma Aldrich, St Louis, MO, USA) was infused via the pancreatic duct. Pancreata were collected in RPMI medium with collagenase and were digested at 37 °C for 12 min. The digested tissue was washed twice in RPMI medium and was filtered through a 500 μm filter to separate the islets from undigested tissues. Islet purification was performed on a discontinuous Ficoll gradient (1.118, 1.096 and 1.037 g/ml, Cellgro by Mediatech Inc., Manassas, VA, USA), respectively, and centrifuged at 625 × g for 16 min without brake. Islets were collected from the aqueous phase between the first and second layer and washed twice with RPMI medium. Finally, islets were incubated overnight in a humidified 5% CO_2_ atmosphere at 37 °C in RPMI 1640 medium containing L-glutamine (Sigma), penicillin-streptomycin (10 mg/ml, Sigma Aldrich) and 10% v/v fetal calf serum (HyClone, Celbio, Logan, UT, USA). Islets were counted and hand-picked under a bright field microscope. Eight hundred islets were transferred to an eppendorf tube and centrifuged at 62 × g for 2 minutes and aspirated in a canula by a Hamilton syringe. Syngenic transplantation was performed in the calf muscle, parallel to the fibula, using needles with a 0.8 mm diameter. To determine the exact number of transplanted islets, the canula was washed with fresh medium which was collected in the eppendorf tube used for preparation of the islets. The exact number of transplanted islets was determined by subtracting the remaining islets in the tube from the initially counted number.

### Radiolabeling of exendin-3

[Lys^40^(DTPA)]-exendin-3 was purchased from Peptide Specialty Laboratories (Heidelberg, Germany). Peptide labeling with In-111 was performed as previously described^14^. Radiochemical purity was determined by ITLC and radiolabeled exendin-3 was purified by solid-phase extraction using a HLB-cartridge (Waters, Etten-Leur, The Netherlands) prior to animal injection as previously reported^14^. All animals were injected with 0.1 μg of exendin-3 corresponding to approximately 15 MBq.

### Binding assay on isolated islets

The binding of radiolabeled exendin-3 to islets was examined on isolated islets from C3H mice. After overnight recovery from isolation procedure, islets were collected and the medium was removed by centrifugation as described above. The islets were diluted in binding buffer (RPMI 1640 containing 0.5% BSA (w/v)) and 100 or 300 islets (triplicate) were added to 24-transwell plates (Corning B.V. Life Sciences, Amsterdam, The Netherlands) and counted under a bright-field microscope (Meyer Instruments, Huston TX, USA). The islets were washed with 200 μl of binding buffer and 1.5 fmol of In-111-exendin-3 in 200 μl of binding buffer were added. Excess of unlabeled exendin-3 (corresponding to 0.62 nmol) was added to 3 additional wells per condition to examine GLP-1R mediated binding. After 4 h incubation at 37 °C the islets were washed twice with binding buffer, the transwells containing the islets were measured in a γ-counter (Wallac 1480 Wizard, Perkin Elmer, Boston, MA, USA) and the bound fraction was determined.

### Imaging procedure and data acquisition

Mice were injected intravenously with 15 MBq of exendin-3 and were scanned 1 hour after injection. Scans were performed with a small animal U-SPECT-II/CT system (MILabs, Utrecht, The Netherlands) with a 1 mm multi-pinhole ultra-high sensitivity mouse collimator for 50 minutes. Mice were scanned after 3 days, 1, 2, 3, 4, 5 or 6 weeks post-transplantation. All mice were scanned twice with 7 days difference, under the same parameters. Immediately after the second SPECT acquisition, mice were euthanized and calf muscles containing the transplant were dissected and were put in 4% paraformaldehyde inside eppendorf® tubes and were scanned *ex vivo* with the same settings. All images were reconstructed with OSEM (3 iterations, 16 subsets, voxel size 0.4 mm) using the U-SPECT-Rec software (MILabs, Utrecht, The Netherlands). CT scans were performed in full/accurate mode or partial/fast mode under 615 μA/s and 65 kV. For quantification of SPECT images, a volume of interest (VOI) was manually drawn around the observed uptake in the transplantation site and the background was subtracted by using the same VOI applied on the contra-lateral calf muscle, which contained no transplant. Data were corrected for the injected dose and converted to Bq by using external standards with known activity scanned and reconstructed with the same settings.

### Histological analyzes

Muscles were fixed in 4% paraformaldehyde and embedded in paraffin after SPECT acquisition. For autoradiography, muscles were sliced into 4 μm sections and were exposed to an imaging plate (Fuji Film BAS-SE 2025, Raytest, Straubenhardt, Germany) for 7 days. Images were visualized with a radioluminography laser imager (Fuji Film BAS 1800 II system, Raytest, Straubenhardt, Germany). Quantitative analysis of the autoradiographical images was performed using AIDA Image Analyzer software (Raytest GmbH, Straubenstadt, Germany). Data were acquired by drawing ROIs around each transplant as well as around the standards with known amount of radioactivity. Subsequently, sections were stained with hematoxylin-eosin (HE) to confirm the presence of pancreatic islets. Finally, for immunohistochemical staining of insulin, consecutive sections underwent antigen retrieval for 10 min in 10 mM citrate pH 6 at 96 °C and blocked with 1% BSA in PBS for 30 minutes and endogenous peroxidase activity was blocked with 3% H_2_O_2_ in PBS for 30 minutes. Staining was performed using anti-insulin rabbit antibody (Santa Cruz Biotechnology, Santa Cruz, CA, USA) diluted 1/500 for 2 hours at room temperature. Sections were incubated with swine anti-rabbit conjugated with peroxidase, for 1 hour at room temperature and washed with PBS. The staining was developed with Bright DAB (Immunologic, Duiven, The Netherlands) and sections were visualized under bright-field microscopy. To normalize the autoradiography measurements by the corresponding transplant surface, the outline of insulin-positive cells of consecutive sections was manually drawn in imageJ (data are expressed as Bq/mm^2^) . To normalize SPECT data by the size of the transplant, the volume was calculated by multiplying the insulin positive surface per level by the distance separating each analyzed section, that is 50 μm. For determination of vascular density, consecutive sections were blocked with 1% BSA in PBS for 30 minutes and stained with anti-insulin guinea-pig antibody (Dako, Heverlee, Belgium) diluted 1/1000 and anti-VEGFR-2 rabbit antibody (Cell signaling, Danvers MA, USA) diluted 1/200 for 2 hours at room temperature. Sections were next incubated with goat anti-guinea pig Alexa 488 (1/200; Life technology, Dallas, TX, USA) and goat anti-rabbit Alexa 594 (1/200; Life technology), respectively, for 1 hour at room temperature. To confirm the expression of the GLP-1R, sections were blocked with 1% BSA in PBS for 30 minutes and stained with anti-GLP-1R rabbit antibody (Abcam, Cambridge, UK) diluted 1/500 for 2 hours at room temperature. Sections were next incubated with goat anti-rabbit Alexa 594 diluted 1/300 for 1 hour at room temperature. Sections were mounted and visualized with an epifluorescence microscope (Axioskop Zeiss, Thornwood, NY, USA).

### Statistical analysis

Measurements are given as means ± SEM. Multiple comparisons were analyzed by one-way ANOVA and Bonferroni post-hoc test, P value < 0.05 was considered statistically significant. Data processing was performed with Graphpad Prism 5 (GraphPad Software, San Diego, CA, USA). Animals that showed no SPECT signal as well as no histological signs of insulin staining, were excluded from the study.

## Additional Information

**How to cite this article**: Eter, W. A. *et al.* Graft revascularization is essential for non-invasive monitoring of transplanted islets with radiolabeled exendin. *Sci. Rep.* **5**, 15521; doi: 10.1038/srep15521 (2015).
